# A Multi-Enzyme Complex That Mitigates Hepatotoxicity, Improves Egg Production and Quality, and Enhances Gut and Liver Health in Laying Hens Exposed to Trace Aflatoxin B_1_

**DOI:** 10.3390/toxins16120517

**Published:** 2024-11-29

**Authors:** Zhuo Chen, Rui Chen, Xin Ma, Wenzi Wu, Qixin Huang, Wenxin Ye, Chulong Wu, Bin Yao, Jianhong Xu, Lichun Qian

**Affiliations:** 1 Key Laboratory of Animal Nutrition and Feed Science in East China, Ministry of Agriculture, College of Animal Sciences, Zhejiang University, Hangzhou 310058, China; 22217071@zju.edu.cn (Z.C.); 22317062@zju.edu.cn (X.M.); 22217014@zju.edu.cn (Q.H.); 2 Hainan Institute of Zhejiang University, Sanya 572025, China; 22317075@zju.edu.cn (R.C.); 22217072@zju.edu.cn (W.W.); 22117080@zju.edu.cn (W.Y.); 22317067@zju.edu.cn (C.W.); 3 State Key Laboratory of Animal Nutrition, Institute of Animal Sciences, Chinese Academy of Agricultural Sciences, No. 2 West Yuanmingyuan Road, Haidian District, Beijing 100193, China; yaobin@caas.cn; 4 Institute of Food Safety and Nutrition, Jiangsu Academy of Agricultural Sciences, Nanjing 210014, China

**Keywords:** AFB1, production, liver damage, gut health, gut microbiota, liver metabolites

## Abstract

Aflatoxin B_1_ is a prevalent secondary hazardous metabolite generated by fungus present in feed ingredients and the surrounding environment: enzymes are currently being recognized as an efficient and promising approach to reducing the associated risks. The objective of this study was to assess the effects of varying doses of enzyme complexes on several parameters in laying hens that were exposed to aflatoxin. During an 8-week experiment, a total of 288 Yukou Jingfen No.6 laying hens were placed into four groups. These groups included a group treated with toxins (CON group) and groups supplemented with compound enzyme complexes at doses of 250 g/t (E1 group), 500 g/t (E2 group), and 1000 g/t (E3 group). The E2 and E3 groups exhibited a statistically significant 2.6% increase in egg production rate compared to the CON group (*p* < 0.05). In addition, the E2 group showed significant improvements in both the feed-to-egg ratio and egg weight (*p* < 0.05). In addition, the E2 and E3 groups showed improved hutch unit and egg white height compared to the control group (*p* < 0.05). The E2 and E3 groups showed a substantial rise in liver health indicators, namely serum alanine transaminase (ALT) and alkaline phosphatase (ALP) activity. On the other hand, malondialdehyde (MDA) was lowered, and total superoxide dismutase (T-SOD) and total antioxidant capacity (T-AOC) were raised. These findings were statistically significant (*p* < 0.05). The E2 and E3 groups showed notable enhancements in intestinal morphology, as evidenced by a rise in villus height and a decrease in crypt depth in all segments of the intestine (*p* < 0.05). Furthermore, analysis of 16S rRNA sequencing revealed that these participants had a higher prevalence and variety of microorganisms in their gut microbiota. More precisely, there was a significant rise in the abundance of *Bacteroidota* and a decline in *Firmicutes* at the level of the phylum. In general, the inclusion of the enzyme complex had advantageous impacts on performance, egg quality, intestinal morphology, intestinal barrier function, and intestinal flora in laying hens. Our results indicate that toxin-degrading enzymes, when used as feed additives, play a significant role in mitigating AFB_1_ contamination in diets and improving the production performance of laying hens.

## 1. Introduction

Mycotoxins are naturally occurring contaminants found in cereals worldwide. Their difficult-to-remove characteristics and their ability to enter the food chain through contaminated cereals, foods, and agricultural by-products pose a significant threat to both animal and human health [1]. Aflatoxin, a common mycotoxin, is produced by the metabolism of various Aspergillus species. The toxicity ranking of aflatoxins is AFB_1_ > AFG_1_ > AFB_2_ > AFG_2_ [2], with AFB_1_ being the most harmful to chickens [3]. Due to its growth characteristics and the storage environment, *Aspergillus* typically infects cereal crops like maize and peanuts [4]. Maize, as the main energy source of poultry feed, accounts for a major proportion of poultry diet, while the allowable level of AFB_1_ in poultry feed is very low, so the level of contamination of poultry feed with AFB_1_ is always higher than the allowable level, resulting in losses in poultry farming [5,6]. AFB_1_ induces hepatic injury in a variety of animals [7,8,9], and in regard to laying hens’ production AFB_1_ leads to decreased egg production and reduced feed conversion [10] and to reduced eggshell thickness and yolk score [11].

AFB_1_ undergoes activation in the liver, transforming into the reactive 8,9-epoxide form. This form attaches to DNA and proteins, resulting in the destruction of the liver structure. Therefore, the liver is recognized as the primary organ affected by AFB_1_ [12]. It has been found that long-term consumption of foods containing excessive amounts of AFB_1_ leads to inflammatory damage in hepatocytes as well as impaired antioxidant functions [12], and that AF-DNA conjugates induce cancer cell proliferation, leading to hepatocellular carcinoma [13].

The bile transports AFB_1_ from the liver to the intestine, exposing intestinal cells to toxic effects. It has been shown that the cycle of circulating absorption of AFB_1_ metabolites can hurt the liver and intestines more severely [14]. For the intestines, AFB_1_ causes the degradation of tight junction proteins that connect the cells of the gut epithelium, leading to intestinal barrier damage, which further affects intestinal morphology [15]. Studies have shown that feeding a diet containing AFB_1_ can reduce the H/D value of the avian intestine [6,16]. Furthermore, AFB_1_ can affect metabolic pathways by disrupting intestinal microbial stability [14], resulting in metabolic diseases. The intestine is the first line of defense against mycotoxins and the first site of absorption by animals. Therefore, we must devise a solution to mitigate the intestinal harm resulting from AFB_1_.

Among the many detoxification methods, biological detoxification is considered the safest and most effective, but ensuring the safety of the product for the organism and its detoxification properties remains the focus of researchers [17]. The enzyme complex utilized in this study was supplied by the Forage Enzyme Engineering Science and Technology Innovation Team of Beijing Animal Husbandry and Veterinary Research Institute, Chinese Academy of Agricultural Sciences, which contains laccase, gibberellinone-degrading enzyme, glucose oxidase, α-galactosidase, and mannanase, which can degrade aflatoxin and protect the liver and intestinal function. This study aimed to evaluate the effects of enzyme complexes on egg production performance, egg quality, liver tissue, intestinal barrier integrity, cecal microbiota, and hepatic metabolites in laying hens exposed to AFB_1_.

## 2. Results

### 2.1. Complex Enzyme Preparation Ameliorated AFB_1_-Induced Decline in Laying Hen Performance

Compared with the control group, the E1 and E3 groups showed a slight increase in laying rate but no significant difference (p>0.05). The E2 group significantly increased the average laying rate in the 8 weeks of the experimental period and ameliorated the decrease in laying rate caused by AFB_1_. No significant difference was observed between the E2 and E3 groups. The FCR of the laying hens was improved by the complex enzyme preparation treatment, but statistically significant differences were not found between the E1 group added at 250 g/t and the control group; the E2 group added at 500 g/t showed significant differences compared to the control and E1 groups; and there was no significant difference between the E3 group added at 1000 g/t and any of the other three groups (Table 1).

### 2.2. Complex Enzyme Preparation Ameliorates AFB_1_-Induced Egg Quality Decline

As shown in Table 2, the compound enzyme preparation had an improvement effect on egg quality, and the E2 and E3 groups with additive amounts of 500 g/t and 1000 g/t significantly improved the hatch unit and egg white height compared with the control group (p<0.05), but the compound enzyme preparation did not have a significant effect on the eggshell strength and eggshell thickness of the eggs.

### 2.3. Complex Enzyme Preparations to Alleviate AFB_1_-Induced Liver Injury

**Figure 1:**
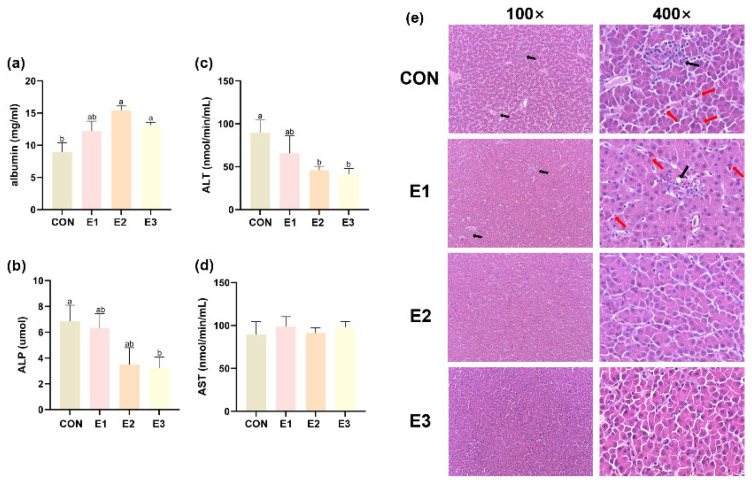
The effects of enzyme complexes on liver tissue morphology and functional abnormalities induced by AFB_1_ in laying hens: (**a**–**d**) The actives of albumin (*n* = 8), ALP, ALT, and AST in serum; values are means ± SEM (*n* = 6). (**e**) Liver tissue HE results (100× and 400×). Partial fragmentation of the nucleus (black arrow), blood infiltration within the sinusoidal spaces (red arrow). The results are represented as mean ± SEM; ^a, b^ represent significant differences between groups (*p* < 0.05).

Histopathological analysis of the liver showed that the liver tissue of the control group had obvious expansion of hepatic sinusoidal stasis and multiple foci of cellular necrosis, while the hepatocytes of the E2 and E3 groups had a tightly arranged structure and no obvious nucleolysis, and the addition of the composite enzyme preparation eased the abnormal changes in the liver tissue and helped the liver to recover. In addition, compared with the control group, the concentration of albumin increased significantly in the E2 and E3 groups and increased slightly in the E1 group (*p* > 0.05), the activity of ALP increased significantly in the E3 group and increased slightly in the E1 and E2 groups, the increase of ALT activity caused by AFB_1_ was inhibited significantly in the E2 and E3 groups, and there was no significant difference between the four groups of AST (Figure 1).

### 2.4. Changes in Antioxidant Activity of the Liver

**Figure 2:**
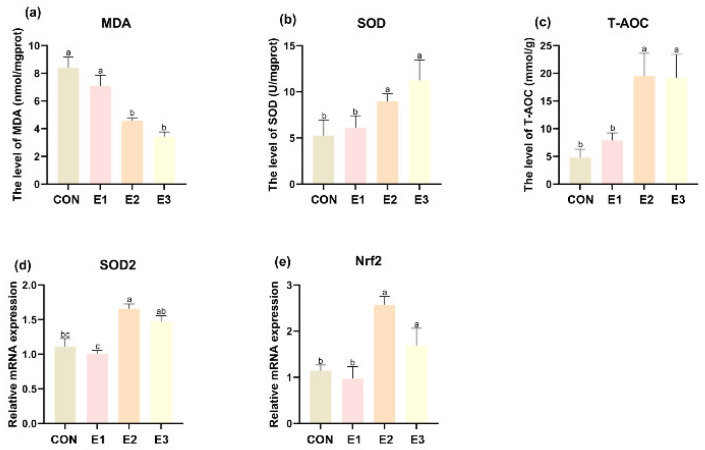
Effects of AFB_1_, AFB_1_+ enzyme complexes treatments on the oxidation and antioxidant levels of MDA, SOD, and T-AOC in the liver (**a**–**c**); values are means ± SEM (*n* = 6). Relative mRNA expression of SOD1 and Nrf2 (**d**,**e**); values are means ± SEM (*n* = 3). ^a–c^ Means with different letters within the same column are significantly different (*p* < 0.05).

To explore the impact of AFB_1_ and AFB_1_ combined with complex enzyme preparation treatments on the antioxidant capacity of laying hen liver, it was observed that MDA levels were significantly decreased, while SOD and T-AOC levels were significantly increased in the E2 and E3 groups compared to both the control and E1 group (Figure 2a–c).

SOD2 expression was markedly higher in the E2 group compared to both the control and E1 groups, while the E3 group showed a slight, though not statistically significant, increase in SOD2 expression compared to the control group (p=0.06). Additionally, Nrf2 expression was significantly elevated in both the E2 and E3 groups when compared to the control and E1 groups (Figure 2e).

### 2.5. The mRNA Expression Levels of Tight Junctions in the Intestinal Epithelial Barrier and Alerations in Intestinal Morphology

**Figure 3:**
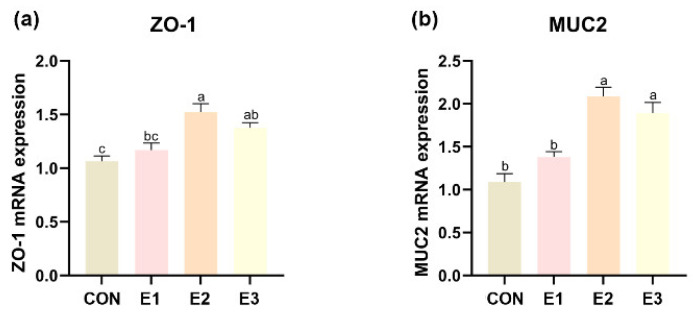
Effects of enzyme complex levels on the mRNA expression levels of tight junctions in jejunum of laying hens feeding in AFB_1_ and complex enzymes at different concentrations: (**a**) zonula occludens 1 (ZO-1); (**b**) mucin 2 (MUC2) (*n* = 4). ^a–c^ Results are expressed as mean ± SEM. Means with different letters within the same column are significantly different (*p* < 0.05).

As shown in Table 3, the addition of complex enzyme preparations ameliorated the destruction of duodenal, jejunal, and ileal structures by AFB_1_. In all intestinal segments, the E2 and E3 groups showed a significant increase in villus height and and a reduction in crypt depth compared to the control group, which resulted in an increased villus height-to-crypt depth ratio (V/C). An exception was observed in the duodenal segment, where the E1 group treatment increased the V/C by decreasing the crypt depth compared to the control group, with no significant differences observed in the other intestinal segments. In the duodenum, there was no notable difference between the E2 and E3 groups; however, in the jejunum and ileum the enhancement of villi was more pronounced in the E3 group, and in the ileum this prominence was also reflected in the V/C. Furthermore, the expression levels of ZO-1 and MUC2 in the jejunum of laying hens was significantly higher in the E2 and E3 groups than in the control group (Figure 3a,b). The results indicate that the addition of 500 g/t and 1000 g/t of enzyme complexes improved AFB_1_-induced intestinal damage.

### 2.6. Diversity and Composition of Cecum Microorganisms

**Figure 4:**
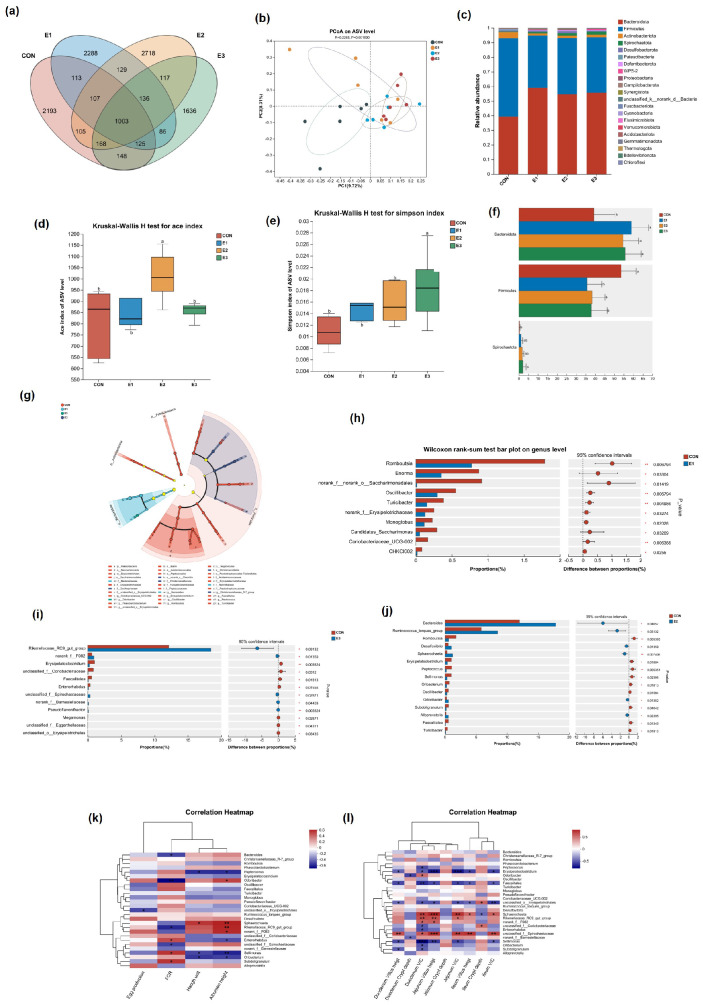
Changes in the gut microbiota of laying hens feeding in AFB_1_ and complex enzymes at different concentrations: (**a**) OTUS analysis. (**b**) β diversity was estimated using PCoA. (**c**) Comparative analysis was performed, to examine the relative abundance of bacteria at the phylum level. The Chao (**d**) and Simpson index (**e**) were used to estimate sample richness and diversity, respectively. (**f**) The distribution of bacterial abundance at the phylum level. (**g**) Cladogram from LEfSe multi−level species difference discriminant analysis (*LDA* > 3), with different color nodes representing those communities significantly enriched in the corresponding phylum, showing significant differences between groups. (**h**) The relative abundance of bacteria communities was also evaluated between the E1 and CON at the genus level, as well as between E2 and CON at the genus level. (**j**) E3 and CON at genus (**i**). (**k**) The heat map illustrates the Spearman correlation between changes in the fecal microbial population and both production performance and egg quality. (**l**) The heat map shows the Spearman correlation between the changes in the fecal microbial population and small intestinal morphology. ^a–c^ Results were expressed as mean ± SEM. Means with different letters within the same column were significantly different (*p* < 0.05). * represents *p* < 0.05, ** represents *p* < 0.01, *** represents *p* < 0.001.

It was found that the number of OTUs in the E1 (3987 OTUs) and E2 (4483 OTUs) groups was higher than that of the CON group (3648 OTUs), with the highest number of OTUs being in the E2 group (Figure 4a). The PCoA plots revealed a notable distinction between the E1, E2, and E3 groups and the CON group (Figure 4b). The alpha diversity index test revealed that the Chao1 index was notably higher in the E2 group, while the Simpson index was significantly elevated in the E3 group (Figure 4d,e). These findings indicate a marked increase in both the total number of species and the community diversity of cecal microorganisms in the E2 group, an increase in species richness in the E1 group, and a significant rise in community diversity in the E3 group. At the phylum level, *Bacteroidota*, *Firmicutes*, and *Actinobacteriota* emerged as predominant phyla (Figure 4c), and by averaging the phyla water in each group, it was found that the addition of the enzyme complex did not change the dominant phyla of the colony but changed the proportion of the dominant phyla in the E1, E2, and E3 groups. *Bacteroidota* increased significantly, *Spirochaetota* increased significantly in the E3 group, *Firmicutes* decreased significantly in the E1, E2, and E3 groups, and the ratio of *Firmicutes*/*Bacteroidetes* decreased.

In addition, the LEfSe analysis (*LDA* > 3) revealed a significant difference in the microbial community structure between the four groups (Figure 4g). The biomarkers of the E1 group were *Bacteroides*, the biomarkers of the E2 group were *Odoribacter*, and the biomarkers of the E3 group were *Christensenellaceae* and *Christensenellaceae_R-7_group*, which are representative groups known to regulate feed efficiency through short-chain fatty acid metabolism. At the genus level, the E2 group had significantly higher abundances of *Odoribacter*, *Ruminococcus_torques_group*, *Desulfovibrio*, *Allobaculum*, and *Sphaerochaeta* and significantly lower abundances of *Erysipelatoclostridium* compared to the CON group. The E3 group had significantly lower abundances of *Sellimonas* and *Enterorhabdus* and significantly higher abundances of *Rikenellaceae_RC9_gut_group* compared to the CON group.

The Spearman correlation analysis between gut microbes and the performance, egg quality, and intestinal villi morphology of laying hens revealed several significant relationships. *Odoribacter* exhibited a strong negative correlation with FCR and a significant positive correlation with protein height. *Sphaerochaeta* was positively correlated with both protein height and haplogroups. *Rikenellaceae_RC9_gut_group* demonstrated a significant negative correlation with FCR and a positive correlation with protein height. *Enterorhabdus* was positively associated with both FCR and haplogroups, while *Erysipelatoclostridium* and *Sellimonas* showed a significant positive correlation with FCR and a negative correlation with haplogroups. *Sellimonas* had a significant positive correlation with FCR and a significant negative correlation with protein height and haplogroups. *Erysipelatoclostridium* and *Sellimonas* showed a significant negative correlation with chorionic villi and the V/C ratio of each intestinal segment, while *Sphaerochaeta* showed a significant positive correlation with chorionic villi and the V/C ratio of each intestinal segment. *Rikenellaceae_RC9_gut_group* exhibited a significant positive correlation with both the duodenal and jejunal V/C ratios as well as with the jejunal villi (Figure 4i). These findings aligned with the observed changes in the abundance of *Erysipelatoclostridium*, *Sellimonas*, *Sphaerochaeta*, and *Rikenellaceae_RC9_gut_group* in the E2 group. Specifically, *Rikenellaceae_RC9_gut_group* and *Sphaerochaeta* are known to metabolically produce short-chain fatty acids, which help protect the intestinal barrier. On the other hand, *Erysipelatoclostridium* and *Sellimonas* have been linked to intestinal inflammation.

### 2.7. Hepatic Metabolism

**Figure 5:**
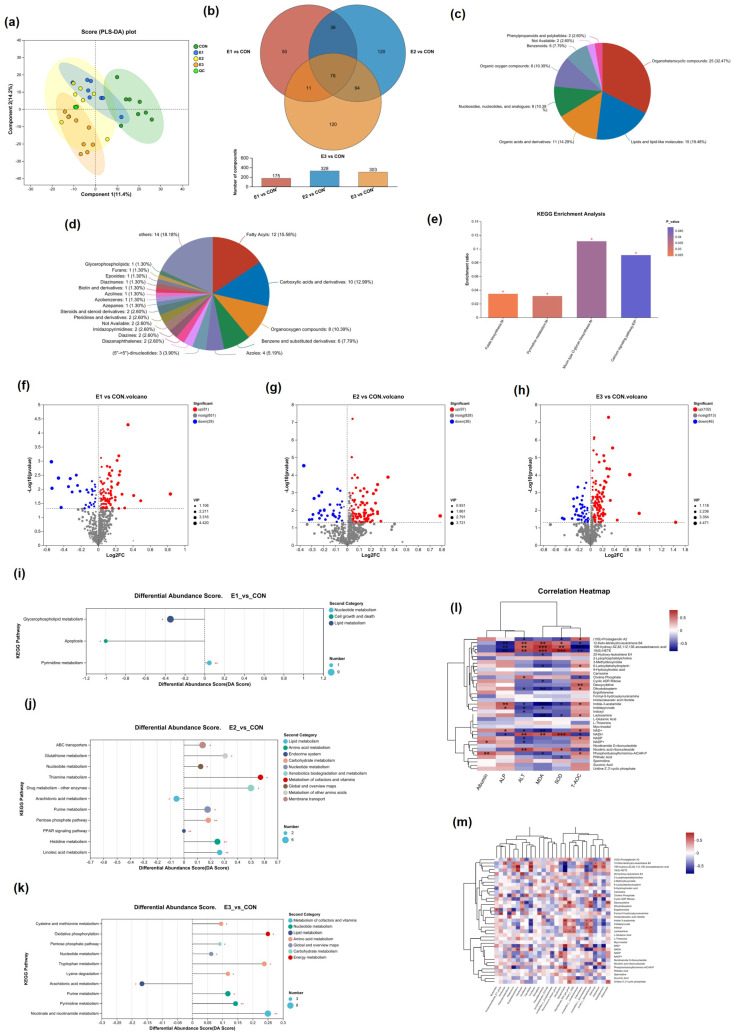
The liver metabo in the comparison groups: (**a**) PLS−DA based on positive ion table. (**b**) Difference Venn diagram of the relative comparison groups. The differential metabolites identified were categorized into 78 distinct biochemical groups. (**c**) Superclass. (**d**) Class. (**e**) KEGG pathways enriched for the 78 differentially expressed metabolites both in the relative comparison group. Volcano plot showing the different fecal metabolites between E1 and CON (**f**), between E2 and CON (**g**), and between E3 and CON (**h**). KEGG enrichment analysis between E1 and CON (**i**), between E2 and CON (**j**), and between E3 and CON (**k**). (**l**) A Spearman correlation analysis was performed, to assess the associations between hepatic metabolites and markers of liver injury and antioxidant capacity. (**m**) Spearman correlation analysis between hepatic metabolites and gut microbiota. * represents *p* < 0.05, ** represents *p* < 0.01, *** represents *p* < 0.001.

The PLS-DA score plot (Figure 5a) showed a clear separation between the CON group and the E1, E2, and E3 groups, suggesting that the compound enzyme treatment greatly ameliorated the liver injury caused by toxin-fed laying hens, and the basic overlap of the QCs indicated that the experiments were of good accuracy and reproducibility. Binary metabolome comparisons were performed for the four groups based on the control group, and according to the Venn diagram in Figure 5b there were 78 common differential metabolites under the three groups treated with different doses of additives, and the analysis of the HMDB compounds revealed that these metabolites were mostly categorized into eight superclasses and 20 classes. Among these superclasses, there were 25 distinct metabolites classified as organic heterocyclic compounds, while 15 were classified as lipids and lipid-like molecules. Subsequent KEGG analysis of the 78 common differential metabolites showed that folate biosynthesis, pyrimidine metabolism, mucin-type O-glycan biosynthesis, and the calcium signaling pathway were the major metabolic pathways significantly altered by the addition of the enzyme complex (Figure 5c). Binary metabolite comparisons were carried out for the four groups based on the control group, and according to the volcano plots (Figure 5f–h) the changes in metabolome data increased with the addition of the complex enzyme preparation, with an increase in the number of significantly altered metabolites.

The CON group was subjected to KEGG analysis with the E1, E2, and E3 groups, respectively (Figure 5i–k). Glycerophospholipid metabolism and apoptosis metabolism were downregulated and pyrimidine metabolism was upregulated in the E1 group, the linoleic acid pathway, histidine metabolism, the PPAR signaling pathway, the pentose phosphate pathway, purine metabolism, drug metabolism–other enzymes, thiamine metabolism in the E2 group, glutathione metabolism, ABC transporters, arachidonic acid metabolism downregulation, E3 group cysteine and methionine metabolism, oxidative phosphorylation metabolism, pentose phosphate pathway, nucleotide metabolism, tryptophan metabolism, lysine degradation, purine metabolism, nicotinic acid and nicotinamide metabolism upregulation, arachidonic acid downregulation.

The Spearman corrected analyses investigated the correlation between liver injury, liver antioxidants, the top 40 gut flora, and liver metabolites. The results showed that 15R-hydroxy-5Z,8Z,11Z,13E-eicosatetraenoic acid, 19(S)-HETE, 12-Keto-tetrahydro-leukotriene B4, NADH, and Nicotinic acid ribonucleoside were significantly positively correlated with MDA, ALT, and SOD and significantly negatively correlated with ALP and T-AOC. NAD^+^ showed a significant positive correlation with both MDA and AL, while it was significantly negatively correlated with ALP and T-AOC. Additionally, indole-3-acetamide was found to be significantly negatively correlated with MDA, ALT, and SOD and significantly positively correlated with ALP and T-AOC. Dihydrobiopterin showed significant negative correlation with MDA, ALT, and SOD and significant positive correlation with T-AOC (Figure 5l); 15R-hydroxy-5Z,8Z,11Z,13E-eicosatetraenoic acid, 19(S)-HETE, NADH, and *Erysipelatoclostridium* and *Faecalitalea* were significantly positively correlated and significantly negatively correlated with *Sphaerochaeta*; 6-Lactoyltetrahydropterin, Dihydrobiopterin, Lactosamine Deoxycytidine, Uridine 2’,3’-cyclic phosphate, and Phthalic Acid were significantly positively correlated with *Rikenellaceae_RC9_gut_group* (Figure 5m).

## 3. Discussion

Mold contamination in animal feed has long been a significant issue, hindering agricultural development and contributing to environmental pollution in the agricultural sector. This study aimed to assess the impact of incorporating complex enzymes on liver protection and intestinal health in livestock and poultry, with the goal of mitigating the harmful effects of aflatoxins in meat, eggs, and milk, thereby reducing their negative impact on human health and the environment.

Excessive AFB_1_ toxin in feeds can damage the reproductive system of poultry [18]. It has been found that AFB_1_ affects egg formation by impairing the mobilization of fats from the liver to the ovaries, thereby decreasing the haustorial unit [16], and that the damage to the reproductive system by AFB_1_ decreases the rate of egg production and egg quality [19], thereby inhibiting chicken performance and causing serious economic losses to the livestock industry. The findings of this study indicate that AFB_1_ levels slightly exceeding the Chinese national standard can negatively impact the production performance of laying hens. However, the inclusion of the complex enzyme preparation appears to mitigate these adverse effects to some extent, leading to an increase in egg production rate, an improvement in FCR, the restoration of overall production performance, and an enhancement in both the Haugh unit and protein height.

The liver is an important detoxification organ for AFB_1_ poisoning [8]. Serum albumin level, ALP, ALT, and AST activity levels are important indicators for assessing liver function, and feeding AFB_1_ increases serum ALT and ALP activity [20,21,22] and decreases serum albumin levels [22]. Histopathological changes in the liver can also be used as a concurrent indicator for assessing in vivo toxicity in AFB_1_-poisoned laying hens. HE-stained sections of the livers of the CON group of laying hens showed severe cellular necrosis, infiltration of inflammatory cells, and dilated hepatic sinusoidal siltation, which is similar to what has been observed in the majority of studies [5,23]. The results of the present study suggest that the addition of the enzyme complex can alleviate AFB_1_-induced liver injury and is most effective when added at 500 g/t.

AFB_1_ interferes with the liver’s antioxidant defense mechanisms, making MDA, T-AOC, and SOD crucial indicators for assessing oxidative stress [21], and it has been shown that feeding diets containing AFB_1_ cause changes in these biochemical markers [24]. The addition of an enzyme complex significantly alleviates the elevated MDA content and reduces the activity of two antioxidant enzymes, T-AOC and SOD, in the liver caused by ABF1; these findings are consistent with those reported in previous studies [22,24]. Furthermore, it has been shown that curcumin can alleviate AFB_1_-induced liver injury by upregulating the Nrf2 pathway [25]. The present study demonstrated that complex enzyme preparations can also alleviate liver injury through this pathway.

The intestinal tract is essential for the digestion and absorption of nutrients, making it vital to ensure the well-being of the intestines for sustainable poultry production. Generally, villus height, crypt depth, and the villus-to-crypt (V/C) ratio are used as indicators of the intestine’s digestive and absorptive efficiency. Typically, an increase in villus height, a decrease in crypt depth, and a higher V/C ratio suggest an enhancement in the intestine’s capacity for digestion and nutrient absorption [26]. To further investigate whether the enzyme complex improved gut health, we examined the mRNA expression of tight junction-associated proteins in the jejunum, as the integrity of the tight junctions in the intestinal epithelium is critical for preventing toxin invasion [27]. The tight junctions are composed of transmembrane proteins (occludin) and auxiliary proteins (occludin band), and ZO-1 (occludin band) is an important membrane protein [28]. MUC2 is the major mucin in the extracellular mucus layer of the intestinal epithelium, which serves as a bacterial nutrient while also allowing for bacterial attachment, contributing to species-specific colonic flora selection [29]. The results showed a significant increase in the expression of ZO-1 and MUC2 in the jejunum of laying hens fed the enzyme complex and an improvement in the intestinal barrier function.

Histological analysis of the cecum microbiota revealed an increase in α diversity in the E2 and E3 groups, and the study showed that higher α diversity indicates a healthier organism [26]. Moreover, by analyzing the changes in microbial phylum levels, it was found that AFB_1_ leads to an increase in the *Firmicutes*/*Bacteroidota* ratio, which is considered to be a biomarker of gastrointestinal tract function and can be indicative of symbiotic conditions in the gastrointestinal tract [30]. In addition, a reduction in the *Firmicutes*/*Bacteroidetes* ratio indicates an alleviation of fatty liver disease, inflammation, and oxidative stress [31,32,33]. The findings demonstrate that the inclusion of the enzyme complex leads to a decrease in the *Firmicutes*/*Bacteroidetes* ratio, potentially contributing to the reduction of inflammation and oxidative stress.

Undigested dietary carbohydrates in the intestine in the presence of *Bacteroides*, *Ruminococcus_torques_group*, *Desulfovibrio*, *Rikenellaceae_RC9_gut_group*, *Sphaerochaeta*, and *Allobaculum* are known to produce short-chain fatty acids, which possess anti-inflammatory properties, modulating the intestinal immune system and protecting the intestinal barrier [31,34,35,36,37]. *Ruminococcus_torques_group* has also been linked to the metabolism of bile acids [36]. In the present study, these short-chain fatty acid-producing bacteria were found to be significantly increased in the E1, E2, and E3 groups. It is worth mentioning in regard to the bacteria *Sellimonas*, *Erysipelatoclostridium*, and *Odoribacter*, which are associated with intestinal inflammation, that the first two of these are increased in intestinal inflammation models [10,38] and that the latter ameliorates inflammatory features by depleting circulating succinate [39]. The present experiment was in line with the former study, with an increase in *Odoribacter* abundance and a decrease in *Sellimonas* abundance in the E2 group and a decrease in *Sellimonas* abundance in the E3 group. The addition of the enzyme complex improved the intestinal inflammatory response, and the Spearman correlation analyses also suggested that bacteria associated with short-chain fatty acid metabolism and those associated with intestinal inflammation were major contributors to the improvement in gut morphology and gut barrier, which was consistent with their roles.

The liver is an important organ for lipid metabolism in animals [40]. This study revealed a relationship between the co-altered differential metabolites in the E1, E2, and E3 groups and lipids, implying that the damage of AFB_1_ to the liver could stem from the disruption of its lipid metabolism. Additionally, the co-altered differential metabolites significantly enhanced the pyrimidine metabolism, a crucial aspect of the animal body. These metabolites serve as structural elements of crucial molecules, crucial for the operation of various cells within the organism [41].

The KEGG enrichment analyses of the E1, E2, and E3 groups, in comparison to the CON group, showed significant downregulation of the arachidonic acid pathway in both the E2 and E3 groups. This pathway was associated with hepatic inflammation, suggesting a reduction in the liver’s inflammatory response. The pentose phosphate pathway was notably upregulated, serving a critical function in providing the precursors necessary for nucleotide and amino acid biosynthesis, producing reducing agents essential for anabolic processes, and mitigating oxidative stress [42]. Moreover, the E2 and E3 groups significantly upregulated various amino acid metabolisms, increased lysine transport affected protein synthesis [43], and histidine metabolism was associated with inflammatory bowel inflammation [44].

A Spearman analysis revealed a significant correlation between the metabolites in the arachidonic acid metabolic pathway and *Sphaerochaeta*, a pathogen associated with liver damage and antioxidants. The compound enzyme preparation may treat intestinal barrier damage and intestinal flora disorders by improving the liver inflammatory response through the intestinal–hepatic axis, and intestinal barrier integrity and healthy intestinal flora, in turn, act on the liver to prevent AFB_1_ from entering the liver through the intestinal–hepatic axis.

Since the enzyme complex is currently not commercially available, as it was formulated based on previous research, commercial production and regulatory approval processes would need to be addressed before large-scale use, potentially increasing costs and delaying application in the field.

## 4. Conclusions

In summary, we demonstrated that the intake of feeding laying hens’ diets with excessive levels of AFB_1_ adversely affected performance and egg quality, with liver damage, disruption of the antioxidant system, disruption of intestinal morphology, and reduction of the mRNA expression levels of tight junctions, which could be mitigated by the addition of complex enzyme preparations. In this study, the first combined analysis of cecum flora and liver metabolites was performed in AFB_1_-intoxicated laying hens, and the results showed that the enzyme complex increased the abundance of SCFAs-producing flora, reduced the abundance of harmful bacteria associated with intestinal inflammation, alleviated intestinal inflammation, and that *Sphaerochaeta* was significantly associated with arachidonic acid metabolism and played an important role in the intestinal–hepatic. The study’s insights on microbial modulation and liver–gut axis protection could inform the creation of probiotics that counteract mycotoxins like AFB1, enhancing resilience in poultry and potentially in broader agricultural contexts. All the results suggest that the enzyme complex can alleviate the negative effects caused by AFB_1_.

## 5. Materials and Methods

### 5.1. Preparation of Aflatoxins

In order to produce aflatoxin, *Aspergillus flavus* (CICC 41668) purchased from China Industrial Microbial Strain Preservation and Management Centre (CIMPSMC) was activated according to the requirements of the purchase instructions and then inoculated in bran slant medium (150 g of bran, 100 mL of double-distilled water) and cultured at 28 °C for 4 days to produce spores. The spores were made into spore suspension and inoculated into the crushed maize that had been autoclaved and cultured at 28 °C for 4 days to obtain fermented maize, and the toxin maize was autoclaved to kill aflatoxins. The Guangzhou Huibiao Inspection and Technology Centre used liquid chromatography–mass spectrometry to quantify aflatoxin content in the fermented maize and obtained 300 kg of fermented maize, which contained 736μg/kg of AFB_1_. The fermented maize was mixed into the diet, according to the ratio in Table 4, to obtain a diet containing 45μg/kg AFB_1_ for the experiment, and the AFB_1_ concentration was rechecked using the Aflatoxin B_1_ detection kit from Shanghai Enzyme Link Biotechnology Co.

### 5.2. Chemicals

The concentrations of total antioxidant capacity (T-AOC, #A005-1-2), malondialdehyde (MDA, #E004-1-1), and total superoxide dismutase (T-SOD, #A003-1-2) were assessed by kits from Nanjing Jiancheng Bioengineering Institute (Nanjing, China). Alanine transaminase (ALT, #ml076532), aspartate aminotransferase (AST, #ml077324), albumin (#ml076981), and the Aflatoxin B_1_ Detection Kit (#YJ036116) were purchased from Shanghai Enzyme Link Biotechnology Co. We measured the OD values at the indicated measurement wavelengths, using an enzyme marker (BioTed, USA).

### 5.3. Experimental Design and Treatment

A total of 288 450-day-old Yukou Jingfen No.6 laying hens were obtained from a local egg farm (Risheng Family Farm, Jiangnan Town, Tonglu, Zhejiang Province, China). The feed given to all the laying hens followed the Chinese standard for poultry farming (MAPRC, 2004.NY/T33-2004). The laying hens were randomly assigned into 4 groups, each consisting of 72 hens, with 6 replicates within each group: CON received a basal diet with 45 μg/kg aflatoxin; E1 group (basal diet with 45 μg/kg aflatoxin and 250 g/T enzyme complexes (containing laccase, gibberellinone-degrading enzyme, glucose oxidase, α-galactosidase, and mannanase)); E2 group (basal diet with 45 μg/kg aflatoxin and 500 g/T enzyme complexes); and E3 group (basal diet with 45 μg/kg aflatoxin and 1000 g/T enzyme complexes). The composition of the basal diets is shown in Table 4. The laying hens were given unrestricted access to their feed, along with fresh water, and were maintained under a 12 h light and 12 h dark cycle. The feeding period lasted for 8 weeks. Approval for all experimental procedures was granted by the Experimental Animal Welfare Ethics Committee of Zhejiang University (Permit No. ZJU20240352). In this experiment, a negative control group was not assigned because prior research had already demonstrated the degradation effect of laccase in the enzyme complex on AFB_1_ toxicity [32]. Based on this established evidence, the focus of this study was on further exploring the efficacy and mechanism of the enzyme complex in degrading AFB_1_ toxicity in laying hens exposed to AFB_1_.

### 5.4. Sample Collection

Throughout the experiment, daily records were kept of the feed intake, egg production, and egg weight of the laying hens for each replicate, and the average egg production and feed conversion ratio (FCR) for the total experimental period were calculated. Feed conversion ratio (FCR) expressed as g feed/g egg was computed by dividing the total feed intake (in grams) by the total egg mass (in grams) for each replicate. At the conclusion of the experiment, 4 egg samples were taken from each replicate (24 egg samples per group) and stored at 4 °C for subsequent determination of egg quality parameters. Eggshell strength, yolk color, protein height, Hartzell unit, and eggshell thickness were determined using an Eggshell Thickness Gauge (ESTG-1, ORKA Technology Ltd., Ramat HaSharon, Israel). After fasting for 24 h before the end of the experiment and euthanizing 12 randomly selected laying hens from each group, the livers were harvested and the cecum samples were promptly frozen at −80 °C for later analysis. Blood samples were drawn from the jugular vein, centrifuged at 3000 rpm for 15 min at 4 °C to isolate the serum, which was then stored at −20 °C for subsequent examination.

### 5.5. Microbiological Analysis of Cecum Samples

The contents from the cecum layer were collected and aseptically transferred under sterile conditions into lyophilized tubes, which were then stored at −80 °C to facilitate subsequent microbiota analysis. DNA extraction from the microbial community was performed using a DNA kit (Omega Biotek, Norcross, GA, USA). The V3–V4 variable region of the 16S rRNA gene was amplified utilizing the universal primers 338F and 806R [8,45]. The PCR products were subsequently sequenced on the Illumina MiSeq platform at Majorbio (Illumina, San Diego, CA, USA) in Shanghai, China. To ensure the quality of the data, QIIME (version 1.9.1) was optimized for PE fragments after processing [41,45], and the representative sequence and abundance information of ASVs (Amplicon Sequence Variants) were obtained.

The Chao species richness, Shannon diversity, and Simpson homogeneity were calculated using the representative sequence and abundance information of ASVs. This study employed the free online tools provided by the Majorbio cloud platform (www.majorbio.com; accessed on 17 November 2024) to evaluate alpha diversity across different groups. Additionally, PCoA was utilized to examine shifts in bacterial community composition, focusing on species differences at the FAMILY and GENUS levels. The Spearman correlation test was used to determine the relationship between key microbiota biomarkers and phenotypic variables, with an FDR-adjusted *p*-value of less than 0.2 being considered statistically significant.

### 5.6. Hepatic Metabolomics Analysis

A non-targeted metabolomic analysis of hepatic tissues from laying hens was conducted using LC-MS. Firstly, the samples were vortexed with cold methanol and extracted by ultrasonication, and the extraction process was repeated three times, after which, the samples were incubated and the supernatant metabolic solvents were collected and analyzed by LC-MS on the UHPLC-Q Exactive platform (Thermo Fisher Scientific, Shanghai, China) of Majorbio Bio-Pharm Technology Co., USA for LC-MS analysis and calibrated using Progenesis QI software (Waters, Milford, CT, USA) to obtain a data matrix [41,45].

To evaluate the system’s stability, quality control (QC) samples were created by combining all samples, then extracting aliquots for analysis at intervals during the analytical run. Principal component analysis indicated that the QC samples clustered tightly together, demonstrating the high reproducibility of the samples.

Similarity of metabolites between samples was examined using PLS-DA, and differences in metabolites between groups were represented by volcano plots. Metabolites that varied between groups were analyzed using a projected importance (*VIP*) value > 1.0 and a *p* value < 0.05. To investigate the potential impact on metabolic pathways following treatment, we conducted metabolic pathway enrichment and topology analysis using the Kyoto Encyclopedia of Genes and Genomes (KEGG, http://www.genome.jp/kegg; accessed on 17 November 2024) database.

### 5.7. Histopathological Analysis

At the conclusion of the experimental phase, tissues from the liver, duodenum, jejunum, and ileum of euthanized laying hens were carefully extracted and preserved in 4% paraformaldehyde for subsequent histopathological analysis. After approximately 48 h of fixation, the tissues were embedded in paraffin and subjected to hematoxylin and eosin staining. Histopathological alterations in the hens were then assessed with a light microscope (Nikon, Tokyo, Japan), and selected tissue sections were captured microscopically. The characteristics observed in the three intestinal segments were quantified using Image J v1.8.0 software (National Institutes of Health, Bethesda, MD, USA).

### 5.8. Real-Time Fluorescence Quantitative PCR

Total RNA extraction from jejunal mucosa and liver was performed using the Trizol method [9]. The concentration of RNA was then determined with a DU 640 Nucleic Acid Spectrophotometer (Beckman Coulter, Inc., 250 S., Brea, CA, USA). Specific primers for the genes zonula occludens 1 (ZO-1), claudin 1, occludin, MUC2, and β-actin are detailed in Table 5. A reaction mixture of 20 μL was prepared for reverse transcription, which included 1000 ng of total RNA and 4 μL of 5× Evo M-MLVRT premix, provided by Hunan Accurate Biotechnology Co., Ltd. Real-time PCR analysis was carried out using the Roche LightCycler 96 system (Roche, Basel, Switzerland). Each reverse transcription reaction was employed as a template in a 20 μL PCR reaction, which included 0.2 mol/L of each primer and SYBR Green premix (Vazyme, Nanjing, China). The reference gene, Chicken β-actin, was used to normalize the data and ensure accurate relative mRNA quantification. The mRNA expression levels were measured by employing the 2^−ΔΔCT^ method [8].

### 5.9. Statistical Analyses

The data analysis was performed using SPSS 29.0 software. Differences among groups were evaluated using one-way ANOVA, followed by the LSD test for post hoc analysis. The experimental data are shown as the mean ± SEM, with p<0.05 representing statistical significance.

## Figures and Tables

**Table 1:** Effects of dietary aflatoxin and enzyme complexes on the performance of laying hens.

Experimental	Egg Production (%)	FCR (g Feed/g Egg Produced)
Control	77.51 ^b^	2.76 ^b^
E1	78.72 ^ab^	2.38 ^b^
E2	81.57 ^a^	2.21 ^a^
E3	80.84 ^ab^	2.33 ^ab^
SEM	0.01	0.75
*p*-value	0.034	0.049

Notes: EP: (hen–day%); FCR: Feed conversion ratio (g feed/g egg produced). ^a, b^ Means with different letters within the same column are significantly different (p<0.05).

**Table 2:** Effects of dietary aflatoxin and enzyme complexes on egg quality.

Experimental	Eggshell Thickness (mm)	Eggshell Strength (kg/cm)	Yolk Colour	Albumen Height	Haugh Unit
Control	0.35	3.07	6.38	5.08 ^b^	69.49 ^c^
E1	0.33	2.97	6.63	5.70 ^b^	74.54 ^bc^
E2	0.34	2.90	6.47	9.01 ^a^	90.95 ^a^
E3	0.35	3.33	6.40	7.87 ^a^	85.50 ^ab^
SEM	0.003	0.10	0.06	0.55	2.99
*p*-value	0.48	0.44	0.52	0.02	0.03

Notes: ^a–c^ Means with different letters within the same column are significantly different (p<0.05).

**Table 3:** Effects of dietary aflatoxin and enzyme complexes on the small intestinal morphology of laying hens.

Item		Treatment			
		**Control**	**E1**	**E2**	**E3**
Duodenum	Villus heigt, mm	1.13 ± 0.10 ^b^	0.90 ± 0.04 ^b^	1.55 ± 0.18 ^a^	1.52 ± 0.07 ^a^
	crypt depth, μm	292.42 ± 24.46 ^a^	145.93 ± 13.99 ^b^	164.83 ± 19.12 ^b^	169.96 ± 12.28 ^b^
	V/C	3.63 ± 0.25 ^c^	6.33 ± 0.46 ^b^	9.46 ± 0.26 ^a^	8.97 ± 0.41 ^a^
Jejunum	Villus heigt, mm	0.99 ± 0.06 ^c^	1.03 ± 0.60 ^c^	1.42 ± 0.08 ^b^	1.76 ± 0.07 ^a^
	crypt depth, μm	240.56 ± 15.39 ^a^	236.44 ± 22.14 ^a^	178.41 ± 10.66 ^b^	211.63 ± 25.44 ^ab^
	V/C	4.21 ± 0.40 ^b^	4.51 ± 0.47 ^b^	8.06 ± 0.57 ^a^	8.77 ± 1.08 ^a^
Ileum	Villus heigt, mm	1.12 ± 0.93 ^c^	0.94 ± 0.05 ^c^	1.46 ± 0.12 ^b^	1.82 ± 0.09 ^a^
	crypt depth, μm	258.00 ± 29.11 ^a^	232.31 ± 18.98 ^ab^	172.45 ± 16.66 ^bc^	164.96 ± 17.49 ^c^
	V/C	4.44 ± 0.31 ^c^	4.19 ± 0.49 ^c^	8.50 ± 0.23 ^b^	11.47 ± 1.22 ^a^

^a–c^ Means with different letters within the same column are significantly different (p<0.05).

**Table 4:** Ingredients and nutrient contents of the basal diet and enzyme complex.

Items	Value
Ingredients, %	
Maize	55.50
Fermented maize	5.80
Soybean meal	24.00
Soybean oil	2.20
Limestone powder	6.50
Fish meal	2.00
Premix	4.00
Total	100.00
Nutrient composition	
Metabolism energy, Mcal/kg	2.82
Crude protein	16.40
Methionine	0.34
Lysine	0.94
Calcium	3.34
Total phosphorus	0.41
Enzyme complex composition, U/g	
Laccase	50
Gibberellinone-degrading enzyme	500
Glucose oxidase	200
α-galactosidase	500
Mannanase	10,000

**Table 5:** Sequence of target gene primers.

Target Gene	Forward Primer	Reverse Primer
β-actin	TGCGTAGGGTTTTGTGTTGG	AACTCCAGACTCCCACACTG
ZO-1	GCTCACAAGCTACGCAAAAA	ACCATCTGCCTTTCCTTCAG
MUC2	GCCTGCCCAGGAAATCAAG	CGACAAGTTTGCTGGCACAT
Nrf2	GAGAAAGCCTTGCTGGCTCA	TGAAGTATCTGTGCTCTGCGAA
SOD2	TACAGCTCAGGTGTCGCTTC	GCGAAGGAACCAAAGTCACG

## Data Availability

The article includes all the relevant data. Unprocessed metabolomics data can be found in the Metabolights public repository, available under the identifier MTBLS10800. Additionally, the raw microbial sequencing data has been uploaded to the NCBI Sequence Read Archive database (SRA accession PRJNA1143184).

## References

[1] Haque M.A., Wang Y., Shen Z., Li X., Saleemi M.K., He C. (2020). Mycotoxin contamination and control strategy in human, domestic animal and poultry: A review. Microb. Pathog..

[2] Jaimez J., Fente C.A., Vazquez B.I., Franco C.M., Cepeda A., Mahuzier G., Prognon P. (2000). Application of the assay of aflatoxins by liquid chromatography with fluorescence detection in food analysis. J. Chromatogr. A.

[3] Chen X., Ishfaq M., Wang J. (2021). Effects of Lactobacillus salivarius supplementation on the growth performance, liver function, meat quality, immune responses and Salmonella Pullorum infection resistance of broilers challenged with Aflatoxin B1. Poult. Sci..

[4] Kumar P., Mahato D.K., Kamle M., Mohanta T.K., Kang S.G. (2017). Aflatoxins: A Global Concern for Food Safety, Human Health and Their Management. Front. Microbiol..

[5] Alm-Eldeen A.A., Basyony M.A., Elfiky N.K., Ghalwash M.M. (2017). Effect of the Egyptian propolis on the hepatic antioxidant defense and pro-apoptotic p53 and anti-apoptotic bcl2 expressions in aflatoxin B1 treated male mice. Biomed. Pharmacother..

[6] Applegate T.J., Schatzmayr G., Pricket K., Troche C., Jiang Z. (2009). Effect of aflatoxin culture on intestinal function and nutrient loss in laying hens. Poult. Sci..

[7] Cao Q., Lin L., Xu T., Lu Y., Zhang C., Yue K., Huang S., Dong H., Jian F. (2021). Aflatoxin B1 alters meat quality associated with oxidative stress, inflammation, and gut-microbiota in sheep. Ecotoxicol. Environ. Saf..

[8] Lin L., Fu P., Chen N., Gao N., Cao Q., Yue K., Xu T., Zhang C., Zhang C., Liu F. (2022). Total flavonoids of Rhizoma Drynariae protect hepatocytes against aflatoxin B1-induced oxidative stress and apoptosis in broiler chickens. Ecotoxicol. Environ. Saf..

[9] Liu M., Li C., Tang H., Gong M., Yue Z., Zhao M., Liu L., Li F. (2022). Dietary lysine supplementation improves growth performance and skeletal muscle development in rabbits fed a low protein diet. J. Anim. Physiol. Anim. Nutr..

[10] Radjabzadeh D., Bosch J.A., Uitterlinden A.G., Zwinderman A.H., Ikram M.A., van Meurs J.B.J., Luik A.I., Nieuwdorp M., Lok A., van Duijn C.M. (2022). Gut microbiome-wide association study of depressive symptoms. Nat. Commun..

[11] Fouad A.M., Ruan D., El-Senousey H.K., Chen W., Jiang S., Zheng C. (2019). Harmful Effects and Control Strategies of Aflatoxin B1 Produced by Aspergillus flavus and Aspergillus parasiticus Strains on Poultry: Review. Toxins.

[12] Uyar A., Yener Z., Dogan A. (2016). Protective effects of Urtica dioica seed extract in aflatoxicosis: Histopathological and biochemical findings. Br. Poult. Sci..

[13] Hathout A.S., Aly S.E. (2014). Biological detoxification of mycotoxins: A review. Ann. Microbiol..

[14] Akinrinmade F.J., Akinrinde A.S., Amid A. (2016). Changes in serum cytokine levels, hepatic and intestinal morphology in aflatoxin B1-induced injury: Modulatory roles of melatonin and flavonoid-rich fractions from Chromolena odorata. Mycotoxin. Res..

[15] Chen J., Lv Z., Cheng Z., Wang T., Li P., Wu A., Nepovimova E., Long M., Wu W., Kuca K. (2021). Bacillus amyloliquefaciens B10 inhibits aflatoxin B1-induced cecal inflammation in mice by regulating their intestinal flora. Food Chem. Toxicol..

[16] Manafi M. (2018). Toxicity of aflatoxin B1 on laying Japanese quails (Coturnix coturnix japonica). J. Appl. Anim. Res..

[17] Adebo O.A., Njobeh P.B., Gbashi S., Nwinyi O.C., Mavumengwana V. (2017). Review on microbial degradation of aflatoxins. Crit. Rev. Food Sci. Nutr..

[18] Raj J., Farkaš H., Jakovčević Z., Vasiljević M., Kumar R., Asrani R.K. (2023). Effects of supplemented multicomponent mycotoxin detoxifying agent in laying hens fed aflatoxin B1 and T2-toxin contaminated feeds. Poult. Sci..

[19] Karimi Torshizi M.A., Sedaghat A. (2023). A consortium of detoxifying bacteria mitigates the aflatoxin B1 toxicosis on performance, health, and blood constituents of laying hens. Poult. Sci..

[20] Kasmani F.B., Torshizi M.A.K., Allameh A., Shariatmadari F. (2012). A Novel Aflatoxin-Binding Bacillus Probiotic: Performance, Serum Biochemistry, and Immunological Parameters in Japanese Quail. Poult. Sci..

[21] Li X., Lv Z., Chen J., Nepovimova E., Long M., Wu W., Kuca K. (2021). Bacillus amyloliquefaciens B10 can alleviate liver apoptosis and oxidative stress induced by aflatoxin B1. Food Chem. Toxicol..

[22] Singh C., Prakash C., Mishra P., Tiwari K.N., Mishra S.K., More R.S., Kumar V., Singh J. (2019). Hepatoprotective efficacy of Premna integrifolia L. leaves against aflatoxin B1-induced toxicity in mice. Toxicon.

[23] Vipin A.V., Raksha R.K., Kurrey N.K., Anu Appaiah K.A., Venkateswaran G. (2017). Protective effects of phenolics rich extract of ginger against Aflatoxin B1-induced oxidative stress and hepatotoxicity. Biomed. Pharmacother..

[24] Abdel-Daim M.M., Abdeen A., Jalouli M., Abdelkader A., Megahed A., Alkahtane A., Almeer R., Alhoshani N.M., Al-Johani N.S., Alkahtani S. (2021). Fucoidan supplementation modulates hepato-renal oxidative stress and DNA damage induced by aflatoxin B1 intoxication in rats. Sci. Total Environ..

[25] Wang H., Muhammad I., Li W., Sun X., Cheng P., Zhang X. (2018). Sensitivity of Arbor Acres broilers and chemoprevention of aflatoxin B1-induced liver injury by curcumin, a natural potent inducer of phase-II enzymes and Nrf2. Environ. Toxicol. Pharmacol..

[26] Miao L., Gong Y., Li H., Xie C., Xu Q., Dong X., Elwan H., Zou X. (2020). Alterations in cecal microbiota and intestinal barrier function of laying hens fed on fluoride supplemented diets. Ecotoxicol. Environ. Saf..

[27] Vaziri N.D., Yuan J., Nazertehrani S., Ni Z., Liu S. (2013). Chronic Kidney Disease Causes Disruption of Gastric and Small Intestinal Epithelial Tight Junction. Am. J. Nephrol..

[28] Wang J., Zhang C., Guo C., Li X. (2019). Chitosan Ameliorates DSS-Induced Ulcerative Colitis Mice by Enhancing Intestinal Barrier Function and Improving Microflora. Int. J. Mol. Sci..

[29] Johansson M.E.V., Larsson J.M.H., Hansson G.C. (2011). The two mucus layers of colon are organized by the MUC2 mucin, whereas the outer layer is a legislator of host–microbial interactions. Proc. Natl. Acad. Sci. USA.

[30] Cheng M., Zhang X., Miao Y., Cao J., Wu Z., Weng P. (2017). The modulatory effect of (-)-epigallocatechin 3-O-(3-O-methyl) gallate (EGCG3"Me) on intestinal microbiota of high fat diet-induced obesity mice model. Food Res. Int..

[31] Ma Q., Li Y., Wang J., Li P., Duan Y., Dai H., An Y., Cheng L., Wang T., Wang C. (2020). Investigation of gut microbiome changes in type 1 diabetic mellitus rats based on high-throughput sequencing. Biomed. Pharmacother..

[32] Wang X., Cui L., Liu M., Qi Z., Luo H., Huang H., Tu T., Qin X., Wang Y., Zhang J. (2024). Theoretical insights into the mechanism underlying aflatoxin B_1_ transformation by the BsCotA-methyl syringate system. Ecotoxico. Environ. Saf..

[33] Yuan C., Fan J., Jiang L., Ye W., Chen Z., Wu W., Huang Q., Qian L. (2023). Integrated Analysis of Gut Microbiome and Liver Metabolome to Evaluate the Effects of Fecal Microbiota Transplantation on Lipopolysaccharide/D-galactosamine-Induced Acute Liver Injury in Mice. Nutrients.

[34] Khan S., Moore R.J., Stanley D., Chousalkar K.K. (2020). The Gut Microbiota of Laying Hens and Its Manipulation with Prebiotics and Probiotics To Enhance Gut Health and Food Safety. Appl. Environ. Microbiol..

[35] Wang R., Bai Y., Yang Y., Wu X., Li R. (2021). A Comparison of Production Performance, Egg Quality, and Cecal Microbiota in Laying Hens Receiving Graded Levels of Vitamin B12. Front. Vet. Sci..

[36] Wu Q., Liang X., Wang K., Lin J., Wang X., Wang P., Zhang Y., Nie Q., Liu H., Zhang Z. (2021). Intestinal hypoxia-inducible factor 2*α* regulates lactate levels to shape the gut microbiome and alter thermogenesis. Cell Metab..

[37] Zhao L. (2013). The gut microbiota and obesity: From correlation to causality. Nat. Rev. Microbiol..

[38] Ning L., Zhou Y.L., Sun H., Zhang Y., Shen C., Wang Z., Xuan B., Zhao Y., Ma Y., Yan Y. (2023). Microbiome and metabolome features in inflammatory bowel disease via multi-omics integration analyses across cohorts. Nat. Commun..

[39] Huber-Ruano I., Calvo E., Mayneris-Perxachs J., Rodríguez-Peña M.M., Ceperuelo-Mallafré V., Cedó L., Núñez-Roa C., Miro-Blanch J., Arnoriaga-Rodríguez M., Balvay A. (2022). Orally administered Odoribacter laneus improves glucose control and inflammatory profile in obese mice by depleting circulating succinate. Microbiome.

[40] Wang Z., Zeng X., Zhang C., Wang Q., Zhang W., Xie J., Chen J., Hu Q., Wang Q., Yang H. (2022). Higher niacin intakes improve the lean meat rate of Ningxiang pigs by regulating lipid metabolism and gut microbiota. Front. Nutr..

[41] Wang X., Guo L., Qin T., Lai P., Jing Y., Zhang Z., Zhou G., Gao P., Ding G. (2024). Effects of X-ray cranial irradiation on metabolomics and intestinal flora in mice. Ecotoxicol. Environ. Saf..

[42] Stincone A., Prigione A., Cramer T., Wamelink M.M.C., Campbell K., Cheung E., Olin-Sandoval V., Grüning N., Krüger A., Alam M.T. (2015). The return of metabolism: Biochemistry and physiology of the pentose phosphate pathway. Biol. Rev..

[43] Khwatenge C.N., Kimathi B.M., Nahashon S.N. (2020). Transcriptome Analysis and Expression of Selected Cationic Amino Acid Transporters in the Liver of Broiler Chicken Fed Diets with Varying Concentrations of Lysine. Int. J. Mol. Sci..

[44] Holeček M. (2020). Histidine in Health and Disease: Metabolism, Physiological Importance, and Use as a Supplement. Nutrients.

[45] Xu P., Dong S., Luo X., Wei B., Zhang C., Ji X., Zhang J., Zhu X., Meng G., Jia B. (2023). Humic acids alleviate aflatoxin B1-induced hepatic injury by reprogramming gut microbiota and absorbing toxin. Ecotoxicol. Environ. Saf..

